# GLCN: Graph-Aware Locality-Enhanced Cross-Modality Re-ID Network

**DOI:** 10.3390/jimaging12010042

**Published:** 2026-01-13

**Authors:** Junjie Cao, Yuhang Yu, Rong Rong, Xing Xie

**Affiliations:** 1 School of Artificial Intelligence and Computer Science, Nantong University, Nantong 226019, China; 2330110408@stmail.ntu.edu.cn (J.C.); 2310110286@stmail.ntu.edu.cn (Y.Y.); rongrong@stmail.ntu.edu.cn (R.R.); 2 Engineering Training Center, Nantong University, Nantong 226019, China

**Keywords:** visible–infrared person re-identification, cross-modality matching, feature alignment, occlusion handling, infrared–visible modality

## Abstract

Cross-modality person re-identification faces challenges such as illumination discrepancies, local occlusions, and inconsistent modality structures, leading to misalignment and sensitivity issues. We propose GLCN, a framework that addresses these problems by enhancing representation learning through locality enhancement, cross-modality structural alignment, and intra-modality compactness. Key components include the Locality-Preserved Cross-branch Fusion (LPCF) module, which combines Local–Positional–Channel Gating (LPCG) for local region and positional sensitivity; Cross-branch Context Interpolated Attention (CCIA) for stable cross-branch consistency; and Graph-Enhanced Center Geometry Alignment (GE-CGA), which aligns class-center similarity structures across modalities to preserve category-level relationships. We also introduce Intra-Modal Prototype Discrepancy Mining Loss (IPDM-Loss) to reduce intra-class variance and improve inter-class separation, thereby creating more compact identity structures in both RGB and IR spaces. Extensive experiments on SYSU-MM01, RegDB, and other benchmarks demonstrate the effectiveness of our approach.

## 1. Introduction

Visible–Infrared Person Re-Identification (VI-ReID) [1,2,3] aims to match pedestrian images across modalities under varying illumination conditions, serving as a key component for all-weather intelligent surveillance. Modern camera systems can automatically switch to infrared mode during nighttime, providing abundant cross-modality data for ReID models. However, the feature distributions of the two modalities differ drastically due to inherent differences between visible and infrared images such as spectral responses, texture structures, and background noise, posing significant challenges to effective cross-modality feature alignment.

In recent years, extensive studies have aimed to mitigate the modality discrepancy in VI-ReID. Existing methods can generally be grouped into two directions: ne line of work attempts to reduce the distribution gap between visible and infrared features by learning a shared feature space or constructing a unified cross-modality mapping [4,5,6,7,8], while the other improves the separability of cross-modality features by incorporating additional information or auxiliary constraints to refine the structure of the learned feature space [9,10,11,12].

**Figure 1:**
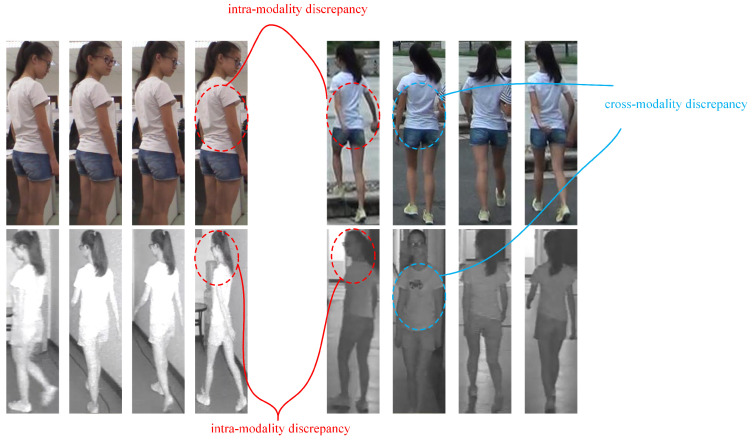
Even within a single modality, images of the same identity can vary significantly due to changes in pose and illumination. When matching across modalities, the discrepancy is further amplified because visible and infrared images are produced by fundamentally different imaging mechanisms, making cross-modality matching considerably more challenging.

Despite the progress brought by these methods, several limitations remain. First, cross-modality feature interaction is insufficient and the complementary semantic information between visible and infrared modalities is not fully exploited. This results in suboptimal cross-modality correlation learning, as illustrated in Figure 1. Second, the modeling of identity–structure relationships is often overlooked. Existing approaches typically lack explicit constraints on stripe-level local features and the geometric relations among identity centers, leading to limited inter-class discriminability. Finally, the issue of intra-modality feature dispersion persists; features belonging to the same identity within a single modality are loosely clustered, which weakens representation stability and degrades retrieval robustness.

To address the issues discussed above, this paper proposes GLCN, a graph-aware locality-enhanced cross-modality network that jointly optimizes cross-modality interaction, structural alignment, and intra-modality compactness. First, at the level of local enhancement and cross-branch interaction, we construct the LPCF module, where LPCG strengthens fine-grained structural cues through local region awareness, positional encoding, and channel selectivity while CCIA introduces a tunable interpolation gate between self-attention and cross-modality contextual attention, enabling stable and controllable semantic interaction across branches. Second, we propose the GE-CGA module, which builds a class-center graph and explicitly aligns the geometric relationship structure at the identity-center level, thereby preserving topology consistency across modalities and enhancing global discriminability. Finally, we introduce the IPDM-Loss, which imposes prototype-guided intra-class compactness and inter-class separation constraints to reduce feature dispersion within each modality and improve identity aggregation. Experiments conducted on the SYSU-MM01, RegDB, and LLCM benchmarks demonstrate that GLCN significantly outperforms state-of-the-art methods across multiple metrics, validating the effectiveness and generality of our cross-modality alignment and locality-enhancement strategies. The main contributions of this work are as follows:

- We propose a unified cross-modality re-identification framework called GLCN which jointly optimizes feature learning through locality enhancement, cross-branch interaction, and structural alignment.
- We design the LPCF module, consisting of LPCG and CCIA, to enhance local structural sensitivity and enable controllable cross-branch semantic interaction.
- We introduce GE-CGA, a graph-enhanced center geometry alignment module that preserves cross-modality topological structures at the class-center level and improves global discriminability.
- We develop the IPDM-Loss, which enhances intra-modality representation compactness and separability through prototype-guided intra-class contraction and inter-class dispersion.

## 2. Related Work

### 2.1. Visible–Infrared Person Re-Identification

VI-ReID aims to match visible and infrared images of the same pedestrian, yet direct feature alignment remains challenging due to the inherent differences between the two modalities in imaging principles, texture distributions, and illumination dependencies. Earlier methods mainly relied on shared feature space mapping, in which metric learning or distribution-level constraints are applied to reduce modality discrepancies. However, these approaches often rely heavily on global representations and struggle to preserve fine-grained discriminative details while enforcing cross-modality consistency. To overcome this limitation, recent studies have introduced local region modeling and multi-level feature fusion strategies. For example, Zhang et al. [6] performed modality compensation at the feature level to achieve cross-modality alignment; Sun et al. [13] enhanced pixel-level local consistency through dense contrastive learning; and Fang et al. [10] explicitly combined local and global features in semantic-level alignment and affinity reasoning. Hua et al. [14] also leveraged vision transformers to model modality-invariant features, focusing on global dependency capture. While this approach excels at learning global representations, it tends to lose finer local details as the network depth increases, making it less effective at capturing and preserving the detailed local information that is crucial for stable cross-modality alignment. Although these methods considerably improve cross-modality alignment, achieving stable and controllable modeling of cross-modality local dependencies without introducing modality specific noise remains an unresolved challenge.

To address this issue, we first construct the LPCF module, which performs structured regional decomposition of features and maintains local spatial constraints, enabling the network to more effectively capture discriminative cues from key body parts during encoding. Meanwhile, LPCF preserves local consistency during cross-branch information interaction, thereby preventing noise interference that may arise from modality discrepancies.

### 2.2. Graph-Based Relational Modeling

Graph structures have been widely adopted in Re-ID to model relationships among samples, body parts, or prototypes, which can effectively enhance identity-level structural representation. Existing graph-based approaches typically perform alignment by constructing explicit node relations; however, most of them treat center features as static nodes and implicitly assume geometric consistency between modalities. In VI-ReID, the substantial modality discrepancy leads to asymmetric geometric distributions of identity centers, while directly aligning these structures often introduces deformation errors. Moreover, graph relations are usually defined within the same semantic level or scale, making it difficult to maintain consistency of cross-modality prototypes in deeper representation spaces. For example, Wu et al. [15] treated visible and infrared modalities as two separate graphs and performed progressive graph matching to explore cross-modality identity correspondence. Similarly, Qiu et al. [16] introduce higher-order structural graphs to model mid-level feature relations and enhance multi-scale structural consistency. Although these approaches extend the role of graph modeling in VI-ReID, they primarily focus on discovering correspondences or modeling mid-level feature graphs while lacking the ability to perform cross-modality geometric and topological alignment at the identity-center graph level.

The proposed GE-CGA module does not align features directly; instead, it aligns the pairwise similarity structures between class centers, constraining the geometric relationship patterns of the two modalities on the identity-level relation graph. This enables the construction of a stable and consistent high-level identity structure across modalities.

### 2.3. Auxiliary Learning in VI-ReID

Another line of research attempts to improve the cross-modality discriminability of VI-ReID through auxiliary learning. The central idea is to introduce additional modality forms, auxiliary image styles, or extra feature branches to provide richer intra-modality variation during training. For instance, Huang et al. [11] enhanced the robustness of cross-modality features using modality-adaptive and invariant decomposition strategies; Miao et al. [12] employed human keypoint estimation as an auxiliary task to strengthen local structural cues; and Zhang et al. [17] integrated multi-modal augmentation branches within a multi-stage auxiliary learning framework to improve representation capability. Hu et al. [18] also followed a similar strategy by introducing an auxiliary modality to enhance its feature extraction capabilities. DJANet employs a dual-branch structure in which one branch processes visible images and the other handles infrared images. This approach aims to reduce modality discrepancies and improve cross-modality matching; however, it often requires additional network branches, image-generation pipelines, or extra modality inputs, which can lead to substantially increased model complexity and even unstable training. Moreover, such methods primarily focus on leveraging complementary information across modalities, yet pay limited attention to whether the intra-modality feature distributions themselves are compact and structurally separable.

In contrast, our approach does not introduce additional modalities or construct extra feature pathways. Instead, we propose IPDM-Loss, which enhances the separability and geometric clarity of the intra-modality feature space by directly acting on the feature structures themselves through multi-level constraints, including intra-class aggregation, inter-class separation, and local–prototype relational consistency. This design provides a more robust representational foundation for subsequent cross-modality alignment. Our strategy represents a lightweight form of auxiliary learning that requires no extra branches, no generative modules, and no expanded input modalities, leading to significantly improved cross-modality matching performance while keeping the inference cost unchanged.

## 3. Methods

### 3.1. Overview

**Figure 2:**
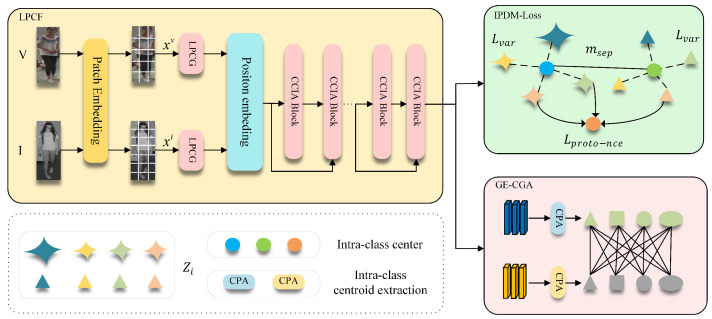
Overall graph-aware locality-enhanced cross-modality Re-ID network architecture.

Our proposed GLCN is built upon a ViT-B/16 [19] backbone, in which visible and infrared images of the same identity are fed into the network to obtain paired features. The overall pipeline of our framework is illustrated in Algorithm 1. On this foundation, we design the LPCF module, in which LPCG first enhances structural and positional sensitivity within local regions and CCIA then performs controllable interpolation between self-attention and cross-branch contextual attention to stably incorporate complementary cross-modality and cross-view information. The interaction between LPCG and CCIA is crucial to the stability and effectiveness of the model. Specifically, LPCG enhances the model’s sensitivity to local structural details, improving its ability to capture fine-grained information within each modality; on the other hand, CCIA addresses the challenge of balancing shallow and deep information by facilitating the interaction between cross-modality self-attention and contextual cross-branch attention, ensuring that both high-level and low-level information are effectively incorporated in the final representation. Meanwhile, the graph-enhanced center geometry alignment module (GE-CGA) constructs a class-center relation graph and aligns its geometric structure across modalities, effectively mitigating semantic structural shifts caused by modality discrepancies. Furthermore, we introduce the Intra-Modal Prototype Discrepancy Mining Loss (IPDM-Loss) to reduce intra-class variance and enlarge inter-class separation, thereby improving the discriminability of features within each modality. Overall, through the coordinated design of local structural modeling, cross-branch information fusion, cross-modality structural alignment, and intra-modality discrepancy regularization, GLCN achieves more robust and discriminative cross-modality representations, as illustrated in Figure 2.
**Algorithm 1:** Overall Training Pipeline of the Proposed GLCN Framework
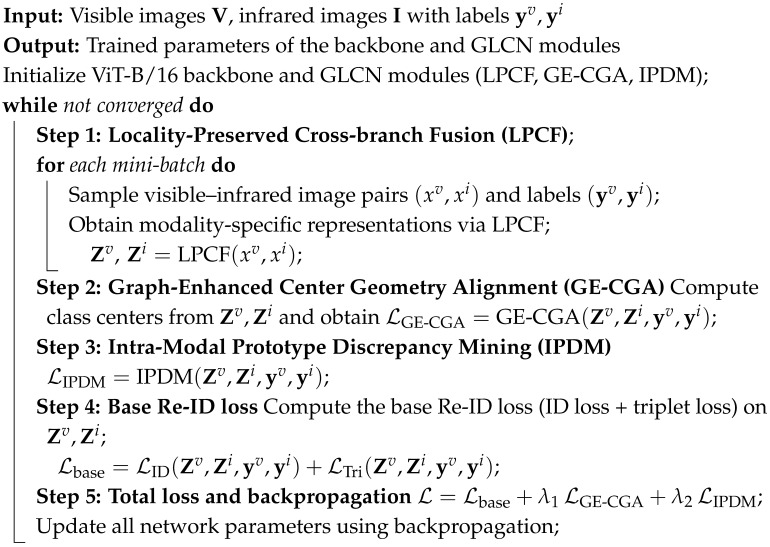


### 3.2. LPCF

**Figure 3:**
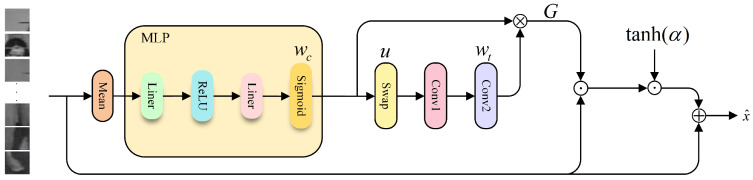
LPCF overall flowchart.

In cross-modality Re-ID, the multi-layer global self-attention in ViT tends to weaken shallow-layer local discriminative semantics, causing deep features to become less sensitive to clothing textures, fine-grained regions, and occlusion details. Recent studies have attempted to enhance locality preservation and local sensitivity; for example, Ni et al. [20] exploited part-aware attention to mine local similarity and improve generalization, Zhang et al. [21] introduced a global–local dual-branch structure to handle occlusions, and Zhou et al. [22] performed local pairwise graph modeling under cross-modality conditions. However, these methods either rely heavily on predefined regions or fixed-granularity partitions or enhance locality only within a single branch, making it difficult to preserve and fuse local details in a stable and progressive manner across different views and modalities. To address this, we propose LPCF, illustrated in Figure 3. LPCG first adaptively preserves local and positional cues at the token level; CCIA then performs controllable interpolation between self-attention and cross-branch contextual attention, enabling effective retention of local information while ensuring stability and consistency during cross-branch feature fusion.

Specifically, given infrared and visible images $V,I\;{\in \;R}^{B\times C\times H\times W}$, we obtain feature maps through patch embedding and flatten them into token sequences $x^{v},x^{i}\in R^{B\times N\times C}$. Prior to feeding the concatenated sequence $x\in R^{2B\times N\times C}$ (stacked from visible and infrared tokens) into the transformer backbone, we introduce the LPCG module to perform joint recalibration across local, positional, and channel dimensions. This strengthens and preserves local discriminative semantics at an early stage, providing a more stable feature foundation for deeper modeling and subsequent cross-branch fusion. We first apply mean pooling along the token dimension and feed the result into a two-layer MLP to generate the channel weights $w_{c}\in {\left(0,1\right)}^{2B\times C}$:

(1) $$w_{c}=\sigma \left(w_{2}\phi \left(w_{1}\cdot mean\left(x\right)+b_{1}\right)+b_{2}\right),$$

where mean(·) denotes averaging along the token dimension, σ(·) is the sigmoid activation function, ϕ(·) represents the ReLU activation, and $w_{1}$,$w_{2}$ together with $b_{1},b_{2}$ are learnable linear projection matrices and their associated bias terms. To further model local positional dependencies among tokens, we first aggregate the input feature map along the channel dimension to obtain a single-channel descriptor and subsequently apply a 1D convolution along the token sequence to capture local neighborhood context. The resulting output is passed through a sigmoid activation to generate the token attention weights $w_{t}\in {\left(0,1\right)}^{2B\times N}$, where the 1D convolution extracts local dependencies across token positions and the sigmoid function ensures normalized attention responses:

(2) $$u=swap\left(x\right)\epsilon R^{2B\times C\times N},$$

(3) $$w_{t}=\sigma \left(Conv2\left(Conv1\left(u\right)\right)\right),$$

where swap(·) exchanges the two groups of sub-channels along the channel dimension, Conv1 is a 1D convolution with a kernel size of 1 for channel compression, and Conv2 is a 1D convolution with a kernel size of 3 for capturing local dependencies along the token sequence. The two attention weights are then fused via an outer product to form the joint weighting matrix $G\in {\left(0,1\right)}^{2B\times N\times C}$, which enables fine-grained local modulation across both positional and channel dimensions. Finally, the recalibrated feature *x* is obtained through residual-style modulation:

(4) $$G=w_{t}⊗w_{c},$$

(5) $$\hat{x}=x+tanh\left(\alpha \right)\cdot Dropout\left(G‘⊙x\right)\in R^{2B\times C\times N},$$

where ⊗ denotes the outer product, ⊙ implemented as element-wise multiplication, $tanh\left(\cdot \right)$ is the hyperbolic tangent activation, and $Dropout\left(\cdot \right)$ randomly deactivates a portion of features during training with a fixed probability. The coefficient α is a learnable scalar parameter. After the LPCF module performs joint token–channel recalibration and local positional enhancement, the resulting feature sequence becomes more locally discriminative and cross-modality stable. We then feed the enhanced sequence $\hat{x}$ into the transformer backbone for cross-layer semantic integration and global modeling. However, as the transformer becomes deeper, shallow-layer semantics tend to be gradually forgotten. To address this issue, we design the CCIA module to enable each block’s self-attention to leverage both the current features and the preserved shallow semantic cues, allowing the attention computation to no longer rely solely on the representation of a single layer. Inspired by the Regional Division (RD) strategy in LAReViT [14], we partition the patch tokens into three non-overlapping regions corresponding to head, trunk, and leg along the vertical spatial dimension. Notably, LAReViT systematically evaluated different numbers of partitions and demonstrated that a three-part division achieves the best performance, striking a favorable balance between local discrimination and global semantic consistency.

After obtaining the global sequence $\hat{x}$, we divide it into three local regions according to the human body structure: the head ${\hat{x}}_{h}$, trunk ${\hat{x}}_{t}$, and legs ${\hat{x}}_{l}$, denoted as ${\hat{x}}_{h},{\hat{x}}_{t},{\hat{x}}_{l}=RD\left(\hat{x}\right)$. We then construct a global branch $\hat{x}=\left[x_{cls},\hat{x}\right]$ and three local branches ${\hat{x}}_{h}=\left[x_{cls},{\hat{x}}_{h}\right]$, ${\hat{x}}_{t}=\left[x_{cls},{\hat{x}}_{t}\right]$, ${\hat{x}}_{l}=\left[x_{cls},{\hat{x}}_{l}\right]$, where $x_{cls}$ the shared class token in the transformer used for aggregating global semantics. The global branch adopts the full positional encoding $E_{pos}$, while the local branches extract their corresponding sub-positional encodings ${\hat{x}}_{h},{\hat{x}}_{t},{\hat{x}}_{l}$ from $E_{pos}$ according to the patch indices associated with ${\hat{x}}_{h}$, ${\hat{x}}_{t}$, and ${\hat{x}}_{l}$, respectively.

(6) $$\hat{x}=\hat{x}+E_{pos}$$

(7) $${\hat{x}}_{h}={\hat{x}}_{h}+\;E_{pos}^{\left(h\right)},{\hat{x}}_{t}={\hat{x}}_{t}+\;E_{pos}^{\left(t\right)}{\hat{x}}_{l}={\hat{x}}_{l}+\;E_{pos}^{\left(l\right)}$$

**Figure 4:**
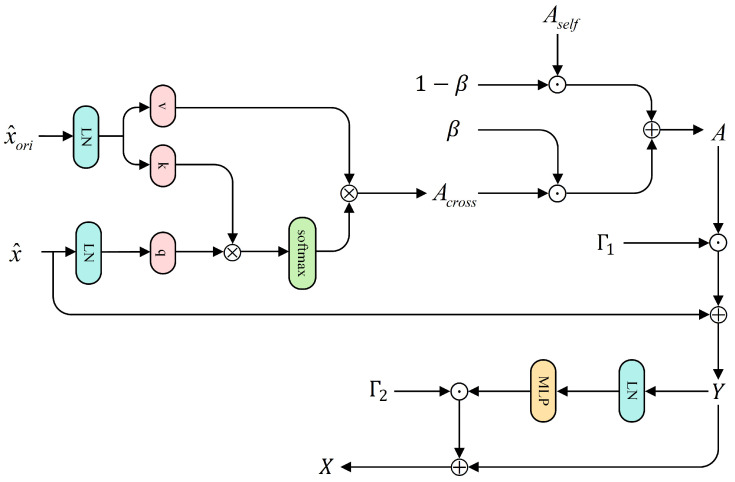
CCIA flowchart.

After constructing the four input branches, we feed the global branch $\hat{x}$ and the three local branches ${\hat{x}}_{h},{\hat{x}}_{t},{\hat{x}}_{l}$ sequentially into the CCIA blocks, which incorporate cross-layer semantic injection, as illustrated in Figure 4.

Specifically, for any branch, let the current-layer input be denoted as $\hat{x}$ and the previous-layer feature as ${\hat{x}}_{ori}$. Note that in the 0-th block, all branches operate purely with self-attention; thus, we set ${\hat{x}}_{ori}=\hat{x}$. Starting from the block 1 onward, the global branch continues to use ${\hat{x}}_{ori}=\hat{x}$ while each of the three local branches sets ${\hat{x}}_{ori}$ to the output of its corresponding branch from the previous layer, thereby injecting cross-layer semantic context. Prior to computing attention, both the current sequence $\hat{x}$ and its reference feature ${\hat{x}}_{ori}$ are individually normalized using layer normalization.

(8) $$\tilde{x}=LN\left(\hat{x}\right),{\tilde{x}}_{\mathrm{ori}}=LN\left({\hat{x}}_{\mathrm{ori}}\right)$$

We then compute the self-attention and cross-branch attention separately:

(9) $$A_{\mathrm{self}}=Att\left(\tilde{x},\tilde{x}\right),A_{\mathrm{cross}}=Att\left(\tilde{x},{\tilde{x}}_{\mathrm{ori}}\right)$$

where the attention operator Att(·) denotes the standard multi-head scaled dot-product attention. For any input *X* and its reference feature $X_{ref}$, we have

(10) $$q=XW_{Q},k=X_{ref}W_{K},v=X_{ref}W_{V},$$

(11) $$Att\left(X,X_{ref}\right)=softmax\left(\frac{qk^{⊤}}{\sqrt{d}}\right)v.$$

When $X_{ref}=X$, the operation corresponds to self-attention, whereas setting $X_{ref}={\tilde{x}}_{ori}$ yields cross-branch attention. We then introduce a learnable scalar gate β to control the proportion of cross-branch contextual injection:

(12) $$\beta =1-exp(-softplus\left(\log\left(1+e^{\beta }\right)\right)),$$

(13) $$A=\left(1-\beta \right)A_{\mathrm{self}}+\beta A_{\mathrm{cross}}.$$

To prevent cross-branch injection from disrupting local semantic consistency, we incorporate LayerScale into the residual path:

(14) $$Y=\hat{x}+\Gamma_{1}\cdot A,$$

(15) $$X=Y+\Gamma_{2}\cdot MLP\left(LN\left(Y\right)\right),$$

where *X* denotes the output feature after the CCIA module and $\Gamma_{1},\Gamma_{2}\in R^{C}$ are learnable scaling parameters that ensure a smooth transition from local self-attention to cross-branch fusion without requiring additional stabilization strategies. After completing attention mixing and inter-layer residual updating, the global branch and the three local branches are normalized using LayerNorm and BatchNorm1d, respectively, yielding the final cross-layer semantic–enhanced features denoted as $z,z_{h},z_{t},z_{l}\epsilon R^{2B\times C}$, collectively referred to as the cross-layer semantic–enhanced representations.

Then, to ensure that the extracted person features are identity-discriminative, we apply the cross-entropy loss ($L_{ce}$) [23] and the triplet loss ($L_{tri}$) [24] to supervise the enhanced representations:

(16) $$L_{base}=L_{ce}+L_{tri}.$$

In summary, LPCF is designed to enhance the model’s ability to capture local details while performing cross-branch information fusion. It achieves this by using the LPCG module, which focuses on enhancing the model’s sensitivity to local structural and positional information, ensuring that fine-grained details are maintained. The CCIA module then facilitates the controlled interpolation of cross-branch contextual information, allowing for stable interaction between the branches. LPCF improves the model’s understanding of local structures and helps mitigate the loss of shallow features during deep-layer processing. This design ensures both global and local features are integrated effectively, enhancing cross-modality alignment.

### 3.3. GE-CGA

In cross-modality Re-ID, the substantial differences between visible and infrared imaging mechanisms often lead to class-center shifts and inconsistent geometric structures for the same identity across the two modalities. In feature space, RGB and IR representations may differ not only in absolute position but also in their inter-class distances and decision boundaries, which become distorted across modalities. Recent approaches attempt to achieve cross-modality alignment by constructing shared or intermediate modalities. For example, Yu et al. [4] introduced a unified modality hub to mitigate distribution discrepancies, while Cheng et al. [25] employed a cross-modality contrastive memory bank to pull positive pairs closer and push negative pairs apart for improved modality consistency. However, these methods generally focus on bringing the two modalities closer at a global distribution level, aligning center positions or overall shapes without ensuring that the relative distances among different identity classes are preserved. To address this limitation, we propose the GE-CGA module, which explicitly constructs a class-center graph and aligns the pairwise geometric structure of class centers across modalities, thereby preserving inter-class topological relationships. This ensures that the resulting feature space still maintains clear identity boundaries and strong discriminative capability when achieving cross-modality alignment.

We first compute the class centers for the visible and infrared modalities within each batch. Let the global features z be split into visible features $X^{r}$ and infrared features $X^{i}$, where $X^{r},X^{i}\in R^{B\times C}$ and their corresponding identity labels are denoted by *y*. The class centers for the two modalities are then defined as

(17) $$c_{y}^{r}=\frac{1}{b}\sum_{x\in \Omega_{y}^{r}}x,c_{y}^{i}=\frac{1}{b}\sum_{x\in \Omega_{y}^{i}}x,$$

where $\Omega_{y}^{r}$ and $\Omega_{y}^{i}$ denote the sets of visible and infrared samples belonging to identity y, respectively. To ensure numerical stability, we apply $\ell_{2}$-normalization to all centers, and the centers of the same identity from the two modalities are encouraged to be close to each other:

(18) $${\hat{c}}_{y}^{r}=\frac{c_{y}^{r}}{\|c_{y}^{r}\|},{\hat{c}}_{y}^{i}=\frac{c_{y}^{i}}{\|c_{y}^{i}\|},$$

(19) $$L_{\mathrm{close}}=\frac{1}{m}\sum_{y}\left(1-\langle {\hat{c}}_{y}^{\;r},{\hat{c}}_{y}^{\;i}\rangle \right).$$

Subsequently, to further construct a class-center contrastive learning objective, the visible and infrared centers of the same identity are treated as mutual positive pairs in the similarity space, while the centers of different identities are regarded as negative samples. This encourages stronger cross-modality identity discrimination:

(20) $$L_{\mathrm{con}}=L_{ce}\left(\frac{{\hat{c}}_{y}^{r}{\left({\hat{c}}_{y}^{i}\right)}^{⊤}}{\tau },y\right)+L_{ce}\left(\frac{{\hat{c}}_{y}^{i}{\left({\hat{c}}_{y}^{r}\right)}^{⊤}}{\tau },y\right)$$

where τ is the temperature coefficient. In addition, to preserve the relative topological structure among classes across modalities, we align the similarity matrices of the two modality-specific class centers:

(21) $$S^{r}={\hat{C}}^{\;r}{\left({\hat{C}}^{\;r}\right)}^{⊤},\;S^{i}={\hat{C}}^{\;i}{\left({\hat{C}}^{\;i}\right)}^{⊤},\;$$

(22) $$L_{\mathrm{geom}}={∥S_{\neg \mathrm{diag}}^{r}-S_{\neg \mathrm{diag}}^{i}∥}_{2}^{2},$$

where $S^{r}$ and $S^{i}$ denote the similarity matrices constructed from all class centers in the RGB and IR modalities, respectively. Because the diagonal elements represent self-similarity and are always equal to 1, they do not convey geometric structure information among different classes. Therefore, we constrain only the non-diagonal entries, denoted as $S_{\neg \mathrm{diag}}^{r}$ and $S_{\neg \mathrm{diag}}^{i}$. The matrices ${\hat{C}}^{\;r}$ and ${\hat{C}}^{\;i}$ are formed by stacking all l2-normalized class centers from the two modalities. Finally, the overall GE-CGA loss is defined as

(23) $$L_{\mathrm{GE}-\mathrm{CGA}}=L_{\mathrm{close}}+L_{\mathrm{con}}+L_{\mathrm{geom}}.$$

Overall, GE-CGA focuses on the alignment of identity centers across modalities. Rather than directly aligning features, it aligns the pairwise similarity structures between class centers by constraining their geometric relationships on the identity-level relation graph. This approach stabilizes the cross-modality alignment by addressing geometric discrepancies between visible and infrared modalities. GE-CGA allows for the construction of a consistent high-level identity structure across modalities, enhancing the robustness of the model without introducing complex architectures or additional memory requirements. This offers a more efficient and effective way of managing cross-modality differences compared to traditional alignment methods.

### 3.4. Intra-Modal Prototype Discrepancy Mining Loss (IPDM-Loss)

Although cross-modality alignment can mitigate the distribution discrepancy between visible and infrared features, intra-modality variations caused by illumination changes, pose differences, and occlusions may still lead to intra-class dispersion and blurred inter-class boundaries within a single modality. To address this issue, we propose the IPDM-Loss, which constructs identity prototypes separately for each modality and explicitly enforces samples of the same identity to move closer to their corresponding modality-specific prototype while introducing inter-class separation among prototypes. This yields more compact intra-class structures and clearer inter-class distinctions. Unlike approaches that rely solely on global contrastive learning or center-based constraints, the IPDM-Loss operates independently within each modality, avoiding the risk of cross-modality alignment adversely affecting the internal structure of a single modality. As a result, it promotes tightly clustered intra-class distributions and preserves well-separated identity boundaries within each modality.

To avoid forming positive pairs between a sample and itself during contrastive learning, we construct a self-excluded prototype representation based on the modality-specific identity centers ${\hat{c}}_{y}$:

(24) $${\hat{c}}_{y}^{-i}=\frac{\sum_{j\in \Omega_{y_{i}}}\left(z_{j}-z_{i}\right)}{\;|\Omega_{y_{i}}|-1}.$$

To enhance the compactness of identity representations within each modality, we unify the prototype-level contrastive constraint and the variance reduction constraint into an intra-modality compactness term. We first construct the prototype-level loss as follows:

(25) $$L_{proto-nce}=L_{CE}\left(\frac{z{\hat{c}}_{y}^{-i}}{\tau },y\right).$$

This term constrains each sample based on relative similarity, encouraging it to be closer to the prototype of its own identity than to other prototypes. This helps to improve discriminative consistency within the modality. In addition, to further compress intra-class distributions and reduce local feature variance, we explicitly minimize the smooth L1 distance [26] between each sample and its self-excluded class center:

(26) $$L_{var}=\frac{1}{n}\sum_{i=1}^{n}{\|z_{i}-{\hat{c}}_{y_{i}}^{-i}\|}_{SL1}.$$

This term provides an absolute geometric contraction constraint, encouraging samples of the same identity to form a more compact distribution within the modality. By increasing the matching score between a sample and its corresponding identity prototype in the prototype-level similarity space, the relative discriminative consistency within each class is enhanced. Meanwhile, $L_{var}$ further contracts intra-class distributions in the absolute feature space by directly minimizing the distance between each sample and its self-excluded class center. The combination of these two terms simultaneously improves both the discriminability and compactness of intra-modality representations.

In addition, to prevent excessive intra-class compression from blurring inter-class boundaries, we impose a margin-based separation constraint between the centers of different identities:

(27) $$L_{\mathrm{sep}}=\frac{1}{K(K-1)}\sum_{k\neq j}\max\;\left(\langle {\hat{c}}_{k},{\hat{c}}_{j}\rangle -m_{\mathrm{sep}},\;0\right)$$

where K denotes the number of identities within the batch, $m_{\mathrm{sep}}$ controls the minimum separable margin to preserve clear decision boundaries between identities, and ${\hat{c}}_{k}$ and ${\hat{c}}_{j}$ represent the mean features of the *k*-th and *j*-th identities within the modality, respectively. Finally, the overall formulation of IPDM-Loss is provided by

(28) $$L_{\mathrm{IPDM}}=L_{\mathrm{proto}-\mathrm{nce}}+L_{\mathrm{var}}+L_{\mathrm{sep}}.$$

### 3.5. Total Loss

Finally, by integrating all components of the model, the total loss is formulated as

(29) $$Loss=L_{base}+\;\lambda_{1}L_{\mathrm{GE}-\mathrm{CGA}}+\;\lambda_{2}L_{\mathrm{IPDM}}.$$

## 4. Experiments

### 4.1. Dataset Introduction

The SYSU-MM01 [27] dataset contains a total of 491 identities. The training set includes 395 identities with 22,258 visible images and 11,909 infrared images, while the test set includes 96 identities with 301 visible images and 3803 infrared images. The RegDB [28] dataset consists of 412 identities, each captured by two overlapping cameras, providing 10 visible (VIS) images and 10 infrared (IR) images per identity. The LLCM [29] dataset contains 713 identities in the training set and 351 identities in the test set.

### 4.2. Evaluation Metrics

During evaluation, we measure the performance of the model by adopting three commonly used metrics in cross-modality ReID: the Cumulative Matching Characteristic (CMC) [30], Mean Average Precision (mAP) [31], and Mean Inverse Negative Penalty(mINP) [1].

### 4.3. Experimental Settings

This study is implemented in PyTorch 2.8.0 and all training and testing are conducted on a single vGPU with 48 GB memory. We adopt ViT-B/16 as the backbone network. All images are resized to 288 × 144; visible images undergo random horizontal flipping, random erasing, and random grayscale augmentation, while infrared images are augmented using independent geometric transformations to maintain modality consistency. During training, each iteration samples four VIS images and four IR images from eight randomly selected identities. We use the AdamW optimizer with a base learning rate of $3e^{-4}$, cosine learning rate decay, and a weight decay of $1e^{-4}$. The model is trained for 40 epochs on all three VI-ReID datasets, with the loss hyperparameters set to $\lambda_{1}$ = 0.3 and $\lambda_{2}$ = 0.5.

For SYSU-MM01, we strictly follow the official single-shot evaluation protocol. Results are reported under both the ALL-search and Indoor-search settings, where ALL-search uses all cameras for query and gallery construction, while Indoor-search restricts both query and gallery samples to indoor cameras only. For RegDB, we adopt the standard evaluation protocol provided by the dataset, conducting experiments under both VIS-to-IR and IR-to-VIS settings, where visible images are used as queries and infrared images as the gallery, and vice versa. For LLCM, we follow the original training and testing splits released with the dataset and report results under both IR-to-VIS and VIS-to-IR retrieval settings. No deviation from the official dataset splits or evaluation protocols is introduced in our experiments.

### 4.4. Comparison with State-of-the-Art Methods

To comprehensively evaluate the effectiveness of the proposed method, we compare our model with a series of state-of-the-art cross-modality person re-identification (VI-ReID) approaches, including PMT [32], CAL [33], DEEN [29], CSMSF [34], CAJ [35], EIFJLF [36], CSC-Net [37], DCPLNet [38], DMPF [39], AGPI2 [40], CSCL [41], MDANet [42], MSCMNet [43], CSDN [44], DDAG [45], AGW [1], DART [46], MIAM [47], HOS-Net [16], LAReViT [14], and DJANet [18]. The comparison is conducted on three challenging image-level VI-ReID benchmarks: SYSU-MM01, RegDB, and LLCM. For SYSU-MM01, we report results under both the ALL-search and Indoor-search settings; for RegDB, evaluations follow the VIS-to-IR and IR-to-VIS testing protocols; and for LLCM, we include both IR-VIS and VIS-IR retrieval tasks.

As shown in Table 1 and Table 2, our method achieves leading performance across all three image-level VI-ReID benchmarks. On SYSU-MM01, our approach is compared with PMT [32], which relies on global transformer-based feature modeling; CAL [33], which focuses on cross-modality alignment enhancement; and DEEN [29], which employs multi-branch fine-grained fusion, achieving Rank-1/mAP scores of 78.0%/74.9% under the ALL-search setting and 86.6%/87.3% under the Indoor-search setting. On RegDB, our approach is compared with MDANet [42], which leverages local detail modeling, and MSCMNet [43], which captures multi-scale semantic correlations; our method reaches 94.2%/91.8% in the VIS→IR setting and 92.6%/90.8% in the IR→VIS setting, demonstrating superior modality alignment and feature consistency. On the more challenging LLCM dataset, which involves low-light conditions and cross-device domain shifts, our method surpasses graph-alignment-based DDAG [45], discriminative-loss-enhanced AGW [1], and the multi-scale local-enhanced transformer architecture LAReViT [14], achieving 56.9%/64.1% for IR→VIS and 66.2%/68.8% for VIS→IR. Overall, while many existing approaches focus on global attention mechanisms, structural labels, or unimodal alignment, our proposed locality enhancement and prototype alignment strategies provide more robust cross-modality representations, particularly in scenarios with significant modality discrepancies.

### 4.5. Ablation Study

We also conduct a systematic ablation study on SYSU-MM01 to evaluate the contribution of each component, as shown in Table 3. Introducing the inter-class structural constraint improves performance from the baseline to 75.1%/71.5%, indicating that preserving relative class relationships helps to alleviate modality discrepancies. Incorporating the CCIA block brings a further improvement to 75.5%/72.0%, while adding local modeling yields a moderate gain of 75.4%/71.6%, demonstrating the robustness of local regions against occlusion and pose variations. Introducing the graph-based structural constraint significantly enhances global alignment capability, resulting in 75.8%/73.2%. Combining CCIA-based interaction with graph modeling further improves the performance to 76.3%/73.2%, and adding the intra-modal compactness constraint boosts the mAP to 73.9%. Finally, when cross-layer local enhancement, graph-based structural alignment, and intra-modal compactness are jointly applied, the model achieves its best performance of 78.0% Rank-1 and 74.9% mAP, demonstrating the effectiveness and complementarity of all components in cross-modality representation learning.

To further investigate the generalization of the proposed components, we additionally report compact ablation results on the RegDB and LLCM datasets in Table 3. RegDB is a relatively small-scale dataset with limited training samples per identity, making it highly sensitive to model capacity and hyperparameter settings. Insufficient model complexity may lead to underfitting, while excessive parameters can easily cause overfitting. Consequently, the performance gains introduced by the proposed modules on RegDB are relatively limited and not always pronounced. In contrast, LLCM provides a larger-scale and more diverse evaluation setting with increased identities and samples, where the proposed model can better accommodate the introduced components. The ablation results on LLCM demonstrate that our method effectively adapts to larger-scale datasets and maintains stable performance improvements, further validating its robustness and generalization capability across datasets of different scales.

Since the class centers in GE-CGA are computed on-the-fly within each mini-batch, we further investigate the sensitivity of GE-CGA to different batch compositions by varying the number of identities per batch while keeping the total batch size fixed. Specifically, we evaluate four configurations with 2, 3, 4, 6 identities per batch, where the corresponding numbers of samples per identity are set to 12, 8, 6, 4, respectively, resulting in a constant batch size of 24. The results are summarized in Table 4, where both the single-run performance and the mean ± standard deviation over multiple trials are reported. Among all settings, the configuration with four identities and six samples per identity achieves the best overall performance, reaching 78.0% Rank-1 accuracy and 74.9% mAP, as well as the highest averaged results (77.07 ± 1.28 Rank-1 and 74.31 ± 1.00 mAP). Notably, although the batch composition varies significantly across different settings, the performance differences remain moderate and do not exhibit noticeable oscillations or instability. This observation indicates that GE-CGA is robust to changes in batch composition and does not overly rely on a specific identity-to-sample ratio. Therefore, the current batch-wise center computation strategy is sufficiently stable in practice, and introducing additional memory banks or momentum-updated centers is unnecessary under our experimental setting.

For a fair and reliable evaluation, we report the performance statistics following dataset-specific evaluation protocols. For SYSU-MM01 and LLCM, all reported results are obtained by averaging over ten independent runs with different random seeds (from 0 to 9), and both the mean and standard deviation are provided to explicitly reflect the stability of the model under different random initializations and data sampling orders. The results are summarized in Table 5. For RegDB, the benchmark itself defines ten fixed testing splits, and the final performance is conventionally reported as the average over these splits. Therefore, the reported results on RegDB already correspond to an averaged performance across multiple trials, and the mean values naturally coincide with the reported numbers.Overall, the consistent performance across multiple random seeds and predefined testing splits demonstrates the robustness and stability of the proposed method under different evaluation settings.

### 4.6. Visualization Analysis

#### 4.6.1. t-SNE Feature Distribution Visualization Analysis

**Figure 5:**
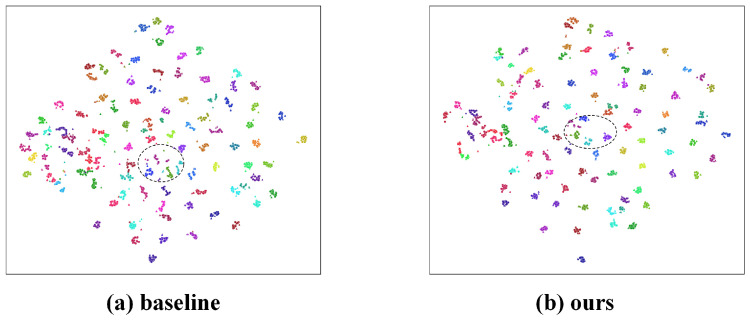
Visualizes the t-SNE results on the SYSU-MM01 test set by randomly sampling about 5000 VIS/IR feature points, covering approximately 90 identities.

Figure 5 presents the t-SNE visualization of high-dimensional feature distributions for the baseline and our method. In the baseline, some identities exhibit loose clusters and severe inter-class mixing, as highlighted by the dashed regions, indicating that cross-modality features are still affected by illumination variations and modality discrepancies. In contrast, our method produces more compact clusters for each identity and significantly reduces overlaps between different classes, resulting in clearer and more separable category boundaries. This demonstrates that the proposed locality enhancement, intra-modality compactness, and graph-structured consistency modules effectively improve the global topological structure of cross-modality features, leading to more natural and discriminative identity clustering.

#### 4.6.2. Retrieval Visualization

**Figure 6:**
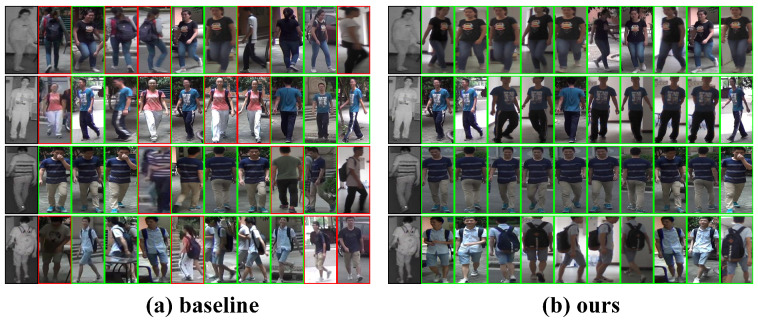
Top-10 cross-modality retrieval visualization. Green boxes indicate correct matches, and red boxes indicate incorrect matches. Compared with the baseline, GLCN retrieves significantly more correct samples and demonstrates markedly improved cross-modality consistency.

Figure 6 illustrates the top-10 retrieval results of the baseline and our method in the cross-modality retrieval task. As shown, the baseline frequently retrieves distractor samples with similar poses but incorrect identities (red boxes), indicating that its cross-modality features still suffer from modality shifts. In contrast, our method consistently retrieves more correct matches, as evidenced by the increased number of green-box results, and maintains high consistency under variations in clothing, pose, and background. It also demonstrates notably stronger robustness when matching between low-light thermal images and visible images. These observations further verify the effectiveness of our approach in modeling cross-modality consistency and identity discrimination.

#### 4.6.3. Visualization of Intra-Class and Inter-Class Distance Distributions

**Figure 7:**
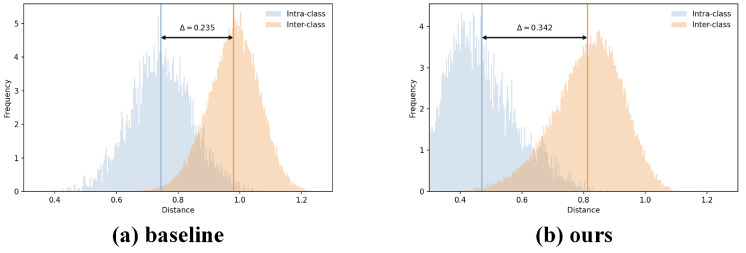
Visualization of intra-class and inter-class distance distributions, where blue indicates intra-class distances and orange indicates inter-class distances. GLCN significantly enlarges the margin Δ, improving feature separability.

As shown in Figure 7, we compare the intra-class and inter-class distance distributions of the baseline and our method in the feature space. The baseline exhibits a large overlap between intra-class and inter-class distributions, with a margin Δ of only 0.235, indicating limited feature discriminability. In contrast, after introducing prototype compression and graph-structure alignment, our method significantly enlarges the separability between the two distributions: the intra-class distances shift leftward overall, while the inter-class distances shift rightward, increasing the margin Δ to 0.342. A larger margin indicates more compact samples within the same class and greater separation between different classes, demonstrating the clear advantage of our approach in enhancing cross-modality discriminability.

#### 4.6.4. Hyperparameter Analysis Figure

**Figure 8:**
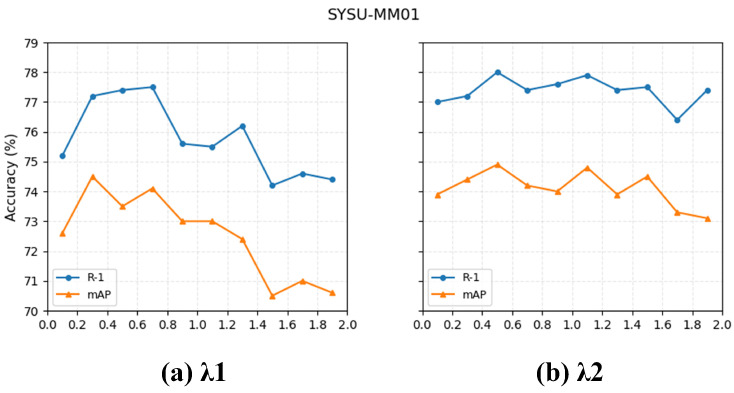
Trends of Rank-1 and mAP under different combinations of $\lambda_{1}$ and $\lambda_{2}$. The best performance is achieved with $\lambda_{1}$ = 0.3 and $\lambda_{2}$ = 0.5.

Figure 8 presents the visualization results of the two hyperparameters $\lambda_{1}$ and $\lambda_{2}$ in our loss function. When fixing $\lambda_{2}$ = 1.0 and increasing $\lambda_{1}$ from 0.1 to 1.9 with a step size of 0.2, both Rank-1 and mAP exhibit a rise-then-fall trend, indicating that a moderate value of $\lambda_{1}$ provides a better balance between constraint strength and feature stability. Similarly, when fixing $\lambda_{1}$ = 0.3 and sweeping $\lambda_{2}$ over the same range, the model shows the most stable performance in the mid-range, with the best results obtained around $\lambda_{2}$ = 0.5. Based on these observations, we adopt $\lambda_{1}$ = 0.3 and $\lambda_{2}$ = 0.5 as the final hyperparameter settings in subsequent experiments to achieve optimal and stable performance.

### 4.7. Efficiency and Computational Cost Analysis

In this section, we present a detailed analysis of the computational efficiency and cost of ours compared to strong baseline methods. Table 6 summarizes the Params, GPU latency (ms/img), and peak GPU memory for ours and several state-of-the-art methods.

As shown in Table 6, ours has a parameter count similar to LAReViT, indicating that the model size is comparable. However, ours outperforms LAReViT on the SYSU-MM01 dataset, demonstrating its superior performance with efficient use of computational resources.

Furthermore, while ours has a smaller parameter count compared to models such as MIP [48] and DEEN, it achieves better results on the SYSU-MM01 dataset. This result highlights that our approach effectively balances model size and accuracy, making it more efficient for Visible-Infrared Person Re-Identification tasks.

## 5. Conclusions

In this work, we address the key challenges of visible–infrared person re-identification, including the loss of local details, significant modality discrepancies, and unstable intra-modality feature distributions. We propose a unified and efficient cross-modality feature learning framework called GLCN. In the proposed framework, the LPCF module is designed to enhance local detail preservation and contextual modeling, the GE-CGA module maintains structural relationships across modality-specific identity classes, and the IPDM-Loss compresses intra-modality variations to improve feature compactness. Ablation studies and visualization analyses further demonstrate the effectiveness of each component in enhancing feature discriminability and cross-modality consistency. Overall, this study provides a concise yet robust solution for cross-modality person re-identification, delivering stable and superior performance under challenging lighting conditions, modality transitions, and real-world environmental disturbances.

## Figures and Tables

**Table 1:** Performance comparison on SYSU-MM01 and RegDB.

Method	Venue	SYSU-MM01		RegDB	
		**ALL Search**	**Indoor Search**	**VIS to IR**	**IR-VIS**
		**R-1/mAP**	**R-1/mAP**	**R-1/mAP**	**R-1/mAP**
PMT [32]	AAAI’23	67.5/65.0	71.7/76.5	84.8/76.6	84.2/75.1
CAL [33]	ICCV’23	74.7/71.7	79.7/83.7	94.5/88.7	93.6/87.6
DEEN [29]	CVPR’23	74.7/71.8	80.3/83.3	91.1/85.1	89.5/83.4
CSMSF [34]	TMM’24	70.6/67.5	76.0/80.2	85.3/76.4	83.9/75.2
CAJ [35]	TPAMI’24	71.5/68.2	76.0/78.4	85.7/79.7	84.9/78.6
EIFJLF [36]	TCSVT’24	72.2/72.8	81.8/84.2	82.0/82.5	82.4/83.2
CSC-Net [37]	TCSVT’24	72.7/69.6	78.6/82.1	91.0/86.4	89.4/85.7
DCPLNet [38]	TII’24	74.0/70.4	78.3/81.9	94.3/87.3	91.7/84.8
DMPF [39]	TNNLS’25	76.4/71.6	82.3/84.9	88.8/81.0	88.9/81.9
AGPI2 [40]	TIFS’25	72.2/70.6	83.5/84.3	89.0/83.9	87.9/83.3
CSCL [41]	TMM’25	75.7/72.1	80.3/83.9	92.2/84.3	89.7/85.1
MDANet [42]	TMM’25	75.8/73.0	80.1/81.8	92.4/82.7	91.0/81.9
CSDN [44]	TMM’25	76.7/73.0	84.5/86.8	95.4/87.7	92.3/85.5
MSCMNet [43]	PR’25	**78.5**/74.2	83.0/85.5	90.4/81.2	87.7/78.2
**Ours**	–	78.0/**74.9**	**86.6/87.3**	**94.2/91.8**	**92.6/90.8**

**Table 2:** Performance comparison on LLCM.

Method	Venue	IR-VIS	VIS-IR
		**R-1/mAP**	**R-1/mAP**
DDAG [45]	ECCV’20	40.3/48.4	48.0/52.3
AGW [1]	TPAMI’22	43.6/51.8	51.5/55.3
DART [46]	CVPR’22	52.2/59.8	60.4/63.2
DEEN [29]	CVPR’23	54.9/62.9	62.5/65.8
MIAM [47]	JVCIR’24	55.4/62.0	62.1/64.5
HOS-Net [16]	AAAI’24	56.4/63.2	64.9/67.9
CSDN [44]	TMM’25	55.8/63.5	63.7/66.5
LAReViT [14]	ACM’25	56.3/63.0	64.1/67.1
DJANet [18]	Vis. Comput.’25	56.8/64.5	66.5/67.5
**Ours**	–	**56.9/64.1**	**66.2/68.8**

**Table 3:** Ablation study of the GLCN framework. The symbol # denotes different module configurations, and - indicates that the corresponding module is not included in that setting.

#	Settings					R-1/mAP		
	Baseline	LPCG	CCIA	GE-CGA	IPDM-Loss	SYSU	LLCM	RegDB
1	✓	-	-	-	-	73.2/69.9	60.8/63.3	92.8/89.8
2	✓	✓	-	-	-	75.1/71.5	61.6/65.0	92.75/88.9
3	✓	-	✓	-	-	75.5/72.0	61.6/65.0	94.3/92.0
4	✓	✓	✓	-	-	75.4/71.6	64.1/66.9	94.1/91.6
5	✓	-	-	✓	-	75.8/73.2	63.0/66.9	94.7/92.3
6	✓	✓	-	✓	-	76.3/73.2	63.7/66.7	94.1/91.7
7	✓	✓	-	✓	✓	76.0/73.9	63.9/66.0	94.7/91.8
8	✓	✓	✓	✓	✓	78.0/74.9	64.5/67.0	94.2/91.8

**Table 4:** Results of the ablation study for GE-CGA module.

Num of Person	Batch	Rank1/Map	Mean (±std)
2	12	76.6/74.9	75.76 ± 0.82/74.19 ± 1.13
3	8	76.8/73.6	74.24 ± 1.39/71.58 ± 1.21
4	6	78.0/74.9	77.07 ± 1.28/74.31 ± 1.00
6	4	75.3/70.6	72.55 ± 1.36/68.90 ± 1.26

**Table 5:** Mean (±std) R-1/mAP results with statistical analysis for different ablation modules. Modules 1 to 8 correspond to the modules listed in Table 3.

#	Mean (±std) R-1/mAP		
	SYSU	LLCM	RegDB
1	72.30 ± 1.08/68.78 ± 0.83	60.81 ± 1.22/63.28 ± 1.14	92.75 ± 2.07/89.78 ± 2.88
2	72.63 ± 1.47/69.57±1.14	61.64 ± 1.30/65.04 ± 1.03	94.34 ± 2.40/91.97 ± 3.13
3	73.42 ± 1.34/69.92 ± 1.04	61.16 ± 1.16/63.72 ± 0.96	94.27 ± 2.51/91.96 ± 3.33
4	73.50 ± 1.10/69.72 ± 0.98	63.08 ± 0.98/66.30 ± 0.69	93.96 ± 2.53/91.64 ± 3.40
5	74.49 ± 1.09/72.06 ± 0.99	64.96 ± 0.80/67.69 ± 0.73	94.74 ± 1.83/79.23 ± 2.44
6	75.56 ± 1.25/72.16 ± 1.14	64.68 ± 0.92/67.52 ± 0.74	94.08 ± 2.10/79.17 ± 2.59
7	75.12 ± 0.95/77.48 ± 1.06	63.88 ± 0.80/66.64 ± 0.74	92.73 ± 1.94/79.05 ± 2.71
8	77.07 ± 1.28/74.31 ± 1.00	64.50 ± 0.94/67.14 ± 0.84	94.22 ± 1.79/91.79 ± 2.42

**Table 6:** Comparison of model performance, parameters, and computational overhead on SYSU-MM01.

Method	Backbone	SYSU	Params	FLOPs	Latency	Peak GPU Men
PMT [32]	Transformer	71.7/76.5	85.64M	31.26G	-	-
DEEN [29]	CNN	80.3/83.3	89.0M	-	-	-
MIP [48]	Transformer	83.5/85.9	103.0M	-	-	-
LAReViT [14]	Transformer	84.2/86.3	85.72M	64.88G	22.41	1500.5
baseline	Transformer	-	85.93M	59.58G	11.92	1473.6
ours	Transformer	86.6/87.3	86.25M	84.64G	20.27	1500.5

## Data Availability

The datasets used in this study (SYSU-MM01, RegDB, and LLCM) are publicly available.

## Abbreviations

The following abbreviations are used in this manuscript:

VI-ReID	Visible–Infrared Person Re-Identification
LPCF	Locality-Preserved Cross-branch Fusion
LPCG	Local–Positional–Channel Gating
CCIA	Cross-Branch Context Interpolation Attention
GE-CGA	Graph-Enhanced Center Geometry Alignment
IPDM	Intra-Modal Prototype Discrepancy Mining
ViT	Vision Transformer
CNN	Convolutional Neural Network
MLP	Multi-Layer Perceptron
GAP	Global Average Pooling
mAP	Mean Average Precision
Rank-1	Rank-1 Accuracy
SYSU-MM01	SYSU Multi-Modal ReID Dataset
RegDB	Visible–Thermal Person ReID Dataset
LLCM	Large-Scale Light-Cloud Modality Dataset
